# Spray Drying of Chokeberry Juice—Antioxidant Phytochemicals Retention in the Obtained Powders versus Energy Consumption of the Process

**DOI:** 10.3390/foods11182898

**Published:** 2022-09-18

**Authors:** Jolanta Gawałek

**Affiliations:** Department of Dairy and Process Engineering, Faculty of Food Science and Nutrition, Poznań University of Life Sciences, Wojska Polskiego 31, 60-624 Poznań, Poland; jolanta.gawalek@up.poznan.pl

**Keywords:** spray drying, chokeberry powder, bioactive properties, polyphenols, antioxidant activity, energy consumption

## Abstract

The production of chokeberry powder, an important functional additive in food, should exhibit both maximization of bioactive properties retention and minimization of energy consumption. The process of spray drying chokeberry juice on a maltodextrin carrier was tested on a semi-technical scale. The research scope included the variability of the inlet air temperature in the range of T = 150–185 °C and concentration of the feed solution in the range of U_d.m_ = 15–45% d.m. The powder yield, energy consumption and bioactive properties of the obtained powders were determined. The highest levels of bioactive properties retention were expressed in total polyphenol content (TPC) and anthocyanin content (AC) and obtained at T = 150 °C and U_d.m_ = 25–30% d.m. However, the most advantageous process parameters in terms of specific energy consumption (SEC) minimization were T = 160–170 °C and U_d.m_ = 30–35% d.m. Analysis of the dependence on SEC versus TPC and SEC versus AC showed that the most favorable drying parameters for chokeberry juice were as follows: inlet air temperature T = 170 °C and feed solution concentration U_d.m_ = 35%. Hence, under such process conditions, chokeberry powders were produced with approx. 3% lower bioactive properties retention (in relation to the maximum values), but with 20.5% lower SEC.

## 1. Introduction

Chokeberry (*Aronia melanocarpa* L.) is a very valuable fruit due to its pro-health properties, in particular in the prevention of many civilization diseases, including some cancers, hypertension, diabetes, atherosclerosis, stomach diseases and age-related deterioration of eyesight [1,2,3,4]. These properties stem from high bioactive phytochemicals content, such as anthocyanins, flavanols, phenolic acids and flavonols [1,5,6]. The aforementioned properties of chokeberry and its various processed forms are widely used in functional foods, which have become increasingly popular in recent years. One of the most commonly used approaches in chokeberry processing is the production of its juice. However, the produced fresh chokeberry juices have a short shelf life and low stability of bioactive properties. Therefore, in the production of functional food with a long shelf life, it becomes necessary to use dehydrated juices. In recent years, the demand for the development of food convenient for quick preparation, including instant food, has been growing steadily [7]. As a consequence, this trend has increased the demand for dehydrated juices. The food in this form requires the use of ingredients produced by various drying methods. In the case of functional food, where the ingredients are a source of bioactivity, it is important that the drying processes of natural ingredients are optimized in order to obtain the highest retention (preservation) of bioactive compounds.

Spray drying is a popular method for this purpose [7,8,9,10,11]; it minimizes losses of bioactive properties in dried plant products compared to other convection drying methods [11]. Previously, several researchers studied spray drying of chokeberry juice and extracts with various carriers [8,9,12,13,14,15,16,17,18]. In the case of spray drying chokeberry juice, Bednarska and Janiszewska-Turak [12], using maltodextrin, gum arabic and their mixtures as carriers, achieved a retention of bioactive compounds in the range of 47.5–97.5, while Gawałek et al., using dextrin [8] and maltodextrin [8,9] carriers, achieved higher retention values (71.3–97.9%). Similarly, in the case of spray drying of polyphenolic chokeberry extracts, Ćujić-Nikolić et al. also achieved high levels of phenolic compound retention (63–97%) using gum arabic [14], maltodextrin and skimmed milk [15] as carriers. Tzatsi and Goula [16], while using mixtures of carriers (maltodextrin-skim milk powder and maltodextrin-whey protein concentrate), attempted to optimize the spray drying parameters of phenolic chokeberry extract and obtained phenolic compounds retention in a wide range (43.77–99.57%).

The results of the presented research show that with the use of appropriate carriers and other process parameters, it is possible to produce spray-dried powders of chokeberry rich in phenolic compounds. However, none of the above studies covered the aspects of energy consumption, and there are no reports in the literature on the relationship between the preservation of bioactive properties during spray drying and the energy consumed by the process. On the other hand, drying processes are one of the most energy-consuming industrial processes, where it is estimated that up to 20% of energy consumption in industry is from these systems [19]. Therefore, attempts to reduce their specific energy consumption (SEC) are very important. This is particularly relevant when considering climate change and the need to reduce the greenhouse effect and adopt a more energy friendly approach. The introduction of the term carbon footprint (CF) allows one to assess the environmental impact of an individual process and product. Minimization of CF in all areas of human activity is a challenge owing to the world’s expansive population. Moreover, the current global political situation has propelled prices of all energy sources, which affects their availability. Therefore, when investigating the optimization of drying processes of natural products while maintaining bioactive properties, it is vital to consider energy consumption.

In this study, the process of spray drying chokeberry juice with a maltodextrin carrier was analyzed on a semi-industrial scale. I examined the trends in energy consumption changes during the process for various feed solution recipes and process parameters. At the same time, changes in bioactive properties during spray drying were analyzed in order to generate optimal chokeberry powders. Determination of changes in bioactive properties in relation to energy consumption during spray drying enabled us to establish optimal drying conditions in terms of minimizing the CF of the process while retaining a high level of bioactive properties. The results of this research may be a basis for the design of energy-efficient processes for the production of chokeberry powders as a promising food ingredient with enhanced antioxidant potential.

## 2. Materials and Methods

### 2.1. Materials

The research material was a concentrate of chokeberry juice (SVZ International B.V., Breda, The Netherlands) with an extract content of 65° Brix. Potato maltodextrin with DE 11 (Roquette Freres, Lestrem, France) was used as the carrier. For all drying processes, the same proportion of chokeberry juice to carrier was used (40% chokeberry juice/60% carrier based on dry matter). After topping up with water, feed solutions were prepared with different dry matter concentrations: 15%, 20%, 25%, 30%, 35%, 40%, and 45%. When testing different inlet air temperatures, the feed solution formula was the same for all drying processes (35% d.m. in water solution, 60% carrier content in dry mass).

### 2.2. Spray Drying

Spray drying of chokeberry juice was carried out using a semi-industrial spray dryer Niro Atomizer type FU 11 DA (Søbork, Denmark) with a diameter of 1.2 m, where the height of the cylindrical part was 0.9 m and the height of the conical bottom was 0.95 m, and the rotary atomizer operation was set at a speed of 12,000 rpm. Additionally, the dryer was equipped with pneumatic hammers to periodically clean the walls of the dryer from accumulated powder. The dryer configured in such a manner on a semi-industrial scale fully reflected the full industrial process. Test drying was carried out at the following inlet air temperatures: 150, 155, 160, 165, 170, 175, 180, and 185 °C. The outlet air temperature was kept constant at 89 ± 1 °C, and the flow rate of the feed solution was in the range of 10–15 L/h as the control variable. The drying air flow rate was maintained at 460 m^3^/h. The spray drying installation was operated under negative pressure, and the powder was collected under the cyclone. In each experiment, the same system and frequency of hammers cleaning the walls of the dryer were used.

When different concentrations of the feed solution were tested, the inlet and outlet temperatures of the drying air were kept constant at 165 °C and 89 ± 1 °C, respectively. The control variable was also the flow rate of the feed solution. The other process parameters remained unchanged when testing different inlet air temperatures.

### 2.3. Moisture Content (MC)

The moisture content of the spray-dried chokeberry juice powder was analyzed by the oven method at 105 °C for 4 h (NFTA Method 2.1.4 with modification of the measurement time [20]).

### 2.4. Powder Yield (PY)

PY was calculated as the ratio of the dry matter of the powder collected under the cyclone after each test spray-drying process to the initial dry matter in feed solution (Equation (1)). After completion of the single spray-drying test, no additional product was collected from the dryer walls.

(1) $$PY=\frac{M_{p}\cdot DM_{p}}{M_{f}\cdot DM_{f}}\cdot 100\;\left[\%\right]$$

*M_p_, M_f_*—mass of powder and feed solution, respectively,
*DM_p_, DM_f_*—percentage of dry matter in the powder and feed solution, respectively.

### 2.5. Total (TWEC) and Effective Water Evaporation Capacity (EWEC)

TWEC in the process of spray drying chokeberry juice was calculated based on the amount of water introduced into the dryer together with the feed solution (Equation (2)). However, EWEC was calculated based on the amount of the obtained powder (Equation (3)).

(2) $$TWEC={\dot{m}}_{f}\cdot \left(WC_{f}-\frac{WC_{p}\cdot (1-WC_{f})}{1-WC_{p}}\right)$$

(3) $$EWEC={\dot{m}}_{p}\cdot \left(\frac{WC_{f}}{1-WC_{f}}-WC_{p}\right)$$

${\dot{m}}_{f},\;{\dot{m}}_{p}$—mass flow rate of the feed solution and powder, respectively,
*WC_f_, WC_p_*—water content in feed solution and powder, respectively.

### 2.6. Specific Energy Consumption (SEC)

SEC of spray drying of the chokeberry juice determined the energy consumption during the process in relation to 1 kg of evaporated water, which was based on the amount of the final product obtained. SEC values for individual drying processes were determined using Equation (4):

(4) $$SEC=\frac{TEC}{EWEC}$$

*TEC* (total energy consumption) was determined using an LE-03d electricity meter (F&F Filipowski Sp. J., Pabianice, Poland). The measurement of energy consumption covered both the drying air heater and fan producing the drying air flow. *EWEC* was calculated using Equation (3).

### 2.7. Total Polyphenol Content (TPC)

The Folin–Ciocalteu method, which was employed in previous studies, was used to determine TPC [9]. Measurements were conducted using a Jasco V630 spectrophotometer (Jasco International Co. Ltd., Tokyo, Japan) by measuring the absorbance at a wavelength of 765 nm. The results were expressed as mg gallic acid equiv. (GA) per 100 g d.m. powder.

### 2.8. Anthocyanin Content (AC)

The anthocyanin content was determined using a Jasco V630 spectrophotometer (Jasco International Co., Ltd., Tokyo, Japan) by the pH differential method according to Giusti and Wrolstad [21]. Absorbance was measured at 520 and 700 nm. Test solutions were diluted with pH 1.0 and 4.5 buffers, and the reference sample was pH 1.0. AC was expressed as cyanidin-3-glucoside (mg Cy-3-G/100 g d.m. of powder).

### 2.9. Statistical Analysis

All determined physicochemical parameters were average values from the measurements taken over a minimum of 3 repetitions. The statistical significance (or not) of the given relationship was verified by one-way analysis of variance (ANOVA) using the Tukey’s test at the significance level of 0.05. Statgraphics 13.1 software was used to process the data.

## 3. Results and Discussion

### 3.1. Moisture Content (MC)

**Figure 1:**
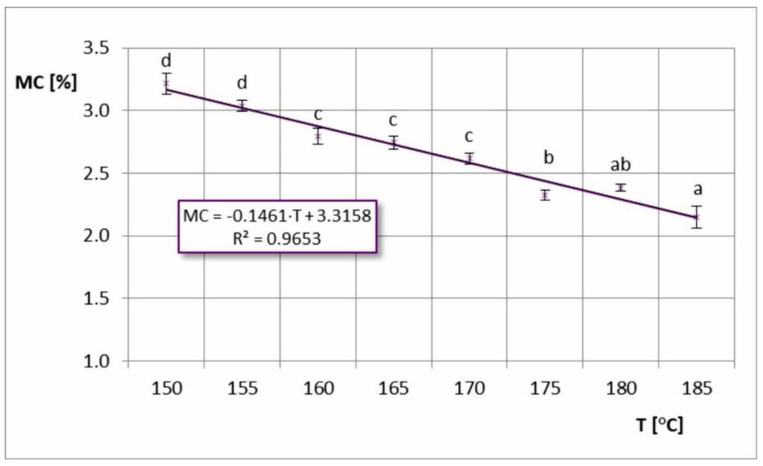
Moisture content (MC) in spray-dried chokeberry juice at different inlet air temperatures (T); (a, b, c, d—different letters show significant differences between mean values (*p* ≤ 0.05)).

The obtained chokeberry powders were characterized by low moisture content values, ranging from 2.15 to 3.22% wb. Figure 1 shows the dependence of the powder’s moisture content on the inlet air temperature. A slight linear trend was observed, where MC decreased with increasing inlet air temperature. This was related to the increase in drying speed at higher temperatures. Additionally, this trend was also confirmed by other studies on spray drying chokeberry juice [8,9,17] and other berries [22,23,24,25,26,27]. However, in the study by Bednarska and Janiszewska-Turak [12], no statistical significance was found for the effect of drying temperature on the moisture content of spray-dried chokeberry juice. Tzatsi and Goula [16], while using mixtures of carriers (maltodextrin-skim milk powder and maltodextrin-whey protein concentrate), obtained different dependences of MC of chokeberry powders in different ranges of inlet air temperatures (for T ≤ 160 °C, increasing the temperature reduced MC, while for T > 160 °C, MC of the powders increased). This trend of change is most likely caused by faster crust formation at higher temperatures and hinders the diffusion of water in the droplet [16].

**Figure 2:**
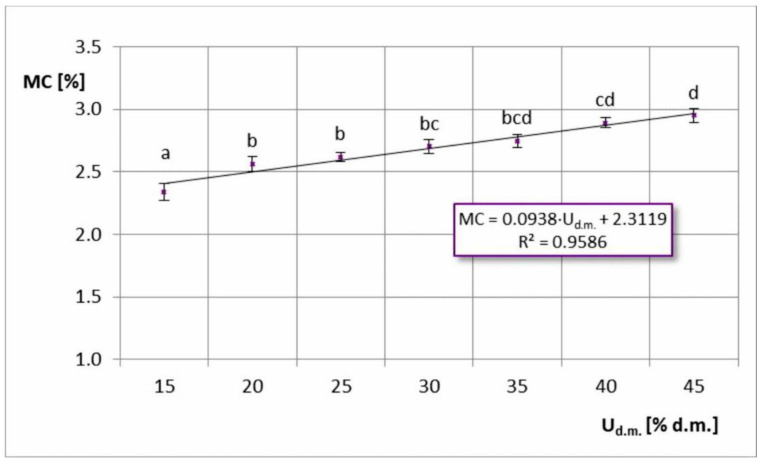
Moisture content (MC) in spray-dried chokeberry juice with different dry matter content in the feed solution (U_d.m._); (a, b, c, d—different letters show significant differences between mean values (*p* ≤ 0.05)).

In the case of dry matter content in the feed solution, a reverse trend was observed (Figure 2), i.e., the moisture content increased with increasing dry matter content in the feed solution. This was due to the fact that spray drying more concentrated feed solutions (which also resulted in higher viscosity) caused the formation of larger particles, which hindered the moisture’s ability to escape from the inside of the particle to the surface. Hence, the spray drying was slower [28,29].

### 3.2. Powder Yield (PY)

**Figure 3:**
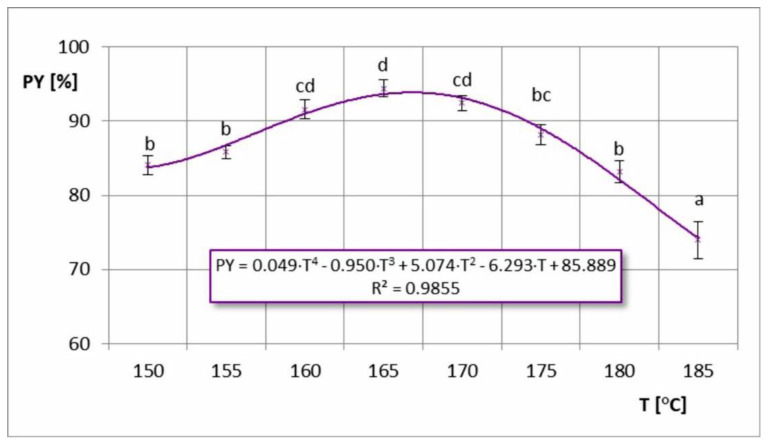
Powder yield (PY) in the process of spray drying chokeberry juice at different inlet air temperatures (T); (a, b, c, d—different letters show significant differences between mean values (*p* ≤ 0.05)).

**Figure 4:**
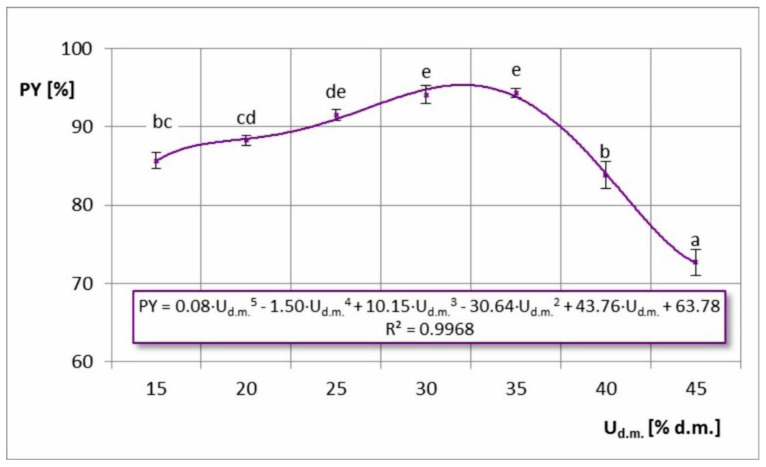
Powder yield (PY) in the process of spray drying chokeberry juice with different dry matter content in the feed solution (U_d.m._); (a, b, c, d, e—different letters show significant differences between mean values (*p* ≤ 0.05)).

Powder yield (PY) is a very important indicator related to the efficiency and economy of the production process. It directly determines the level of raw material losses during the process, which should be as low as possible. In industrial practice, it is expected to maximize powder yield at least above 90%, and preferably above 97% [7]. In the case of the dependence of the powder yield on the inlet air temperature, its graphic image indicates the presence of a maximum (Figure 3). The highest PY values (91.6–94.4%) were obtained during drying temperatures of T = 160–170 °C. Drying at other temperatures reduced the amount of the final product, which may stem from the thermoplastic properties of the chokeberry powder. These properties enhanced the stickiness of the dryer at low temperatures due to insufficient drying of the powder, and at higher temperatures due to exceeding the sticky-point temperature of the powder. In previous studies related to spray drying of chokeberry juice [8,9], the powder yield increased with increasing drying temperature (without an obtained maximum). However, the temperature range was narrower and included lower temperatures (T = 150–170 °C). Similar trends were also obtained by other researchers in the case of spray drying juices of other berries, such as acai juice [23] and mulberry juice [24]. However, a contrasting (decreasing) relationship was obtained by Sharifi et al. [30] in spray drying of barberry juice. In this study, only two temperatures were tested (160 and 180 °C); hence, it was not possible to observe the maximum temperature. It was assumed that at 180 °C (as in the case of spray drying of chokeberry juice), a substantial effect on the powder sticking would decrease PY value. Vidović et al. [17] also noted a maximum PY in spray drying of chokeberry juice, but at a lower temperature (T = 140 °C). However, it was not a semi-industrial process, but only conducted on a laboratory scale. A similar dependence (with maximum) was also observed for the dry matter content in the feed solution, where the optimum powder yield was in the range of 25–35% d.m. (Figure 4). At lower concentrations of feed solution, the greatest product losses were probably related to the increase in fine dust escaping through the cyclone. The lower the concentration of the feed solution, the smaller the formed particles, and not all of them needed to be effectively separated in the cyclone [28]. In the case of higher concentrations of dry matter in the solution, sticking to the equipment certainly had a greater impact on product yield. At higher feed solution concentrations, the centrifugal atomizer created larger particles, which required longer drying times. As a result, a large proportion of the particles that were not completely dried stuck to the walls of the dryer and remained in the equipment. Some of them, after drying on the walls and being hit with shaking hammers, fell off and went to the cyclone, but a large amount remained permanently.

### 3.3. Total (TWEC) and Effective Water Evaporation Capacity (EWEC)

**Figure 5:**
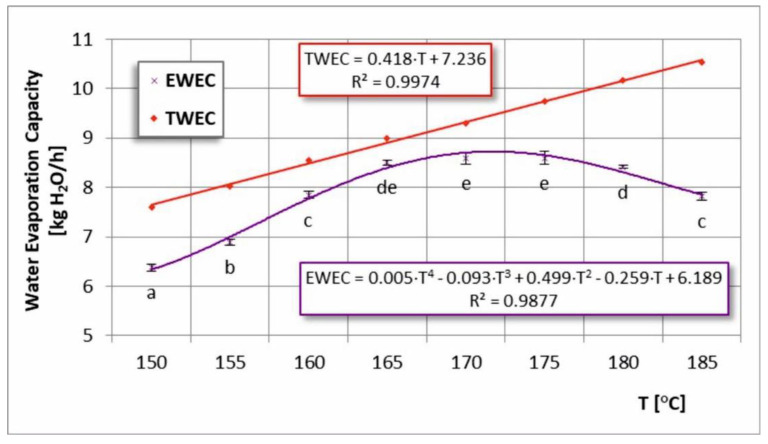
Total (TWEC) and effective water evaporation capacity (EWEC) in the process of spray drying chokeberry juice at different inlet air temperatures (T); (a, b, c, d, e—different letters show significant differences between mean values (*p* ≤ 0.05)).

Figure 5 shows the dependence of the total (TWEC) and effective water evaporation efficiency (EWEC) on the inlet air temperature. An increase in the inlet air temperature promoted an increase in TWEC during the spray-drying process of chokeberry juice. TWEC was determined from the flow rate of the feed solution, which followed a linear increasing trend. EWEC was calculated on the basis of the actual amount of product obtained and had a non-linear trend and was closest to TWEC in the temperature range of T = 160–170 °C. This was determined as the most effective range related to water evaporation, which also correlated with the obtained powder yield results (Figure 3). Beyond this range, both graphs diverged more from each other due to greater sticking to the equipment at too low and high drying temperatures and a greater decrease in water evaporation efficiency observed at higher temperatures.

**Figure 6:**
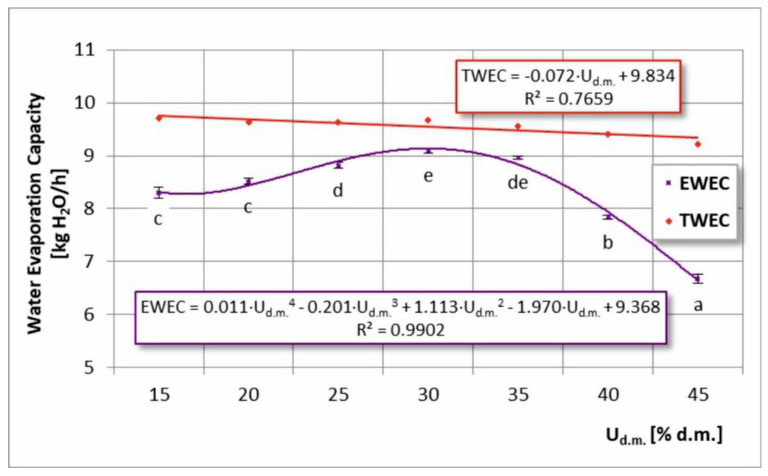
Total (TWEC) and effective water evaporation capacity (EWEC) in the process of spray drying chokeberry juice with different dry matter content in the feed solution (U_d.m._); (a, b, c, d, e—different letters show significant differences between mean values (*p* ≤ 0.05)).

A similar trend in TWEC and EWEC plots was noted when examining the effect of dry matter content in the feed solution (Figure 6). It was possible to determine the most effective range, which was 25–35% of the dry matter content. Contrary to the temperature dependence, the feed solution concentration dependence was decreasing.

### 3.4. Specific Energy Consumption (SEC)

**Figure 7:**
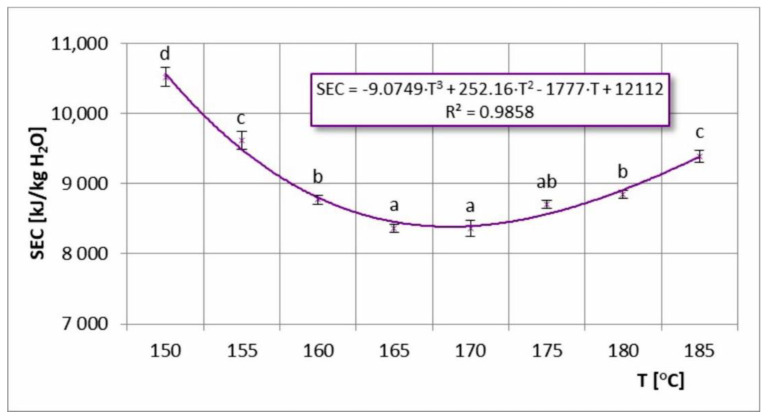
Specific energy consumption (SEC) in the process of spray drying chokeberry juice at different inlet air temperatures (T); (a, b, c, d—different letters show significant differences between mean values (*p* ≤ 0.05)).

SEC at various inlet air temperatures is shown in Figure 7. At 150–185 °C temperature range, SEC values of 8364–10,524 kJ/kg of water evaporated were achieved. Baker and McKenzie [31] determined that the SEC for industrial spray dryers usually ranges from 3000–20,000 kJ/kg H_2_O. For pilot spray dryers, these values can be several times higher. Barańska et al. [32], for spray drying buckwheat honey with maltodextrin on a pilot scale, observed SEC of 24,800 kJ/kg H_2_O. Al-Mansour et al. [33] achieved much lower SEC in the range of 3000–5500 kJ/kg H_2_O when drying coffee extract on a pilot scale. In this case, very low values were related to very wide differences in inlet and outlet air temperature (ΔT = 100–170 °C). In my study, the highest energy consumption rates were observed at the lowest inlet temperatures, as it was impossible to achieve an effective high temperature difference ΔT during drying (at a constant outlet air temperature). Lowering the outlet temperature of the drying air did not improve the energy efficiency of the process due to under-drying of the product and sticking to the dryer walls. This promoted lower EWEC values. Increasing the inlet air temperature to 165–170 °C resulted in the lowest SEC values being obtained, which indicated the optimal minimum of the temperature range. By further increasing the inlet air temperature (T > 170 °C), a slight increase in SEC occurred, which was more gradual than that observed for 150–165 °C. Higher drying temperatures increased the temperature of the product and, at the same time, its tendency to stick, which lowered EWEC and increased SEC.

**Figure 8:**
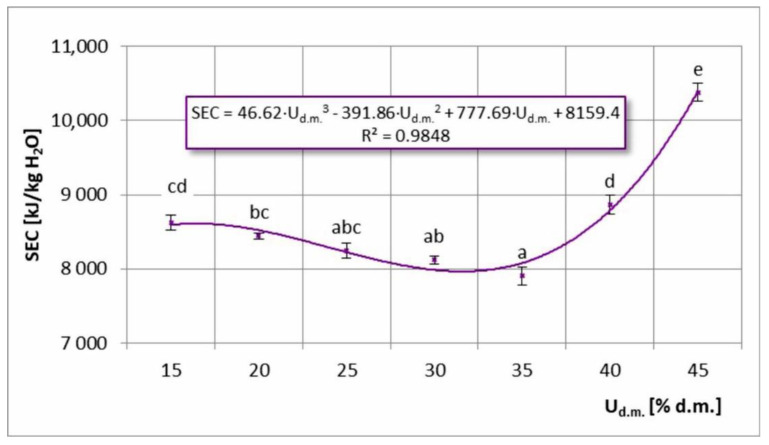
Specific energy consumption (SEC) in the process of spray drying chokeberry juice with different dry matter content in the feed solution (U_d.m._); (a, b, c, d, e—different letters show significant differences between mean values (*p* ≤ 0.05)).

SEC for different values of dry matter content in the feed solution is shown in Figure 8. The course of the graph was comparable to that of SEC at different drying temperatures. In this case, the minimum was observed at 35% d.m. Higher SEC values for higher feed solution concentrations were associated with the formation of larger particles when spraying the feed solution, which required longer drying times. This phenomenon also increased the tendency of the powder to stick and lowered the energy efficiency of the process (increasing SEC ratings). In the case of feed solutions with a lower concentration, smaller particles were formed during atomization, which were lost at the outlet of the cyclone, reducing product yield. This in turn led to lower EWECs and higher SEC ratings.

### 3.5. Total Polyphenol Content (TPC) and TPC–SEC Relationship

**Figure 9:**
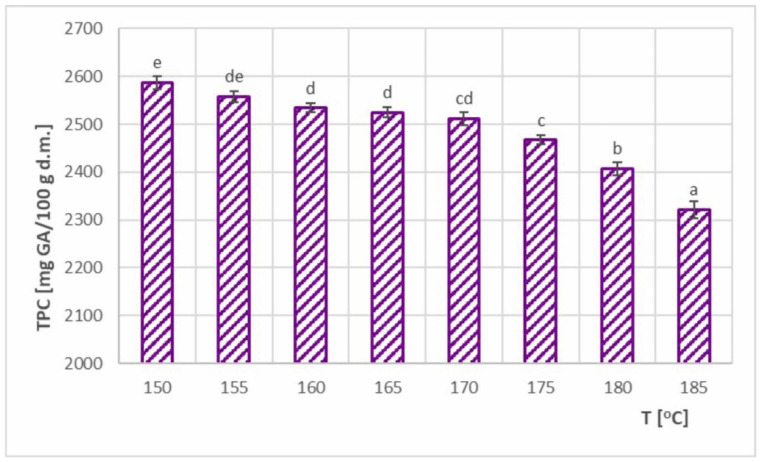
Total polyphenol content (TPC) in spray-dried chokeberry juice at different inlet air temperatures (T); (a, b, c, d, e—different letters show significant differences between mean values (*p* ≤ 0.05)).

The total polyphenol content (TPC) at different inlet air temperatures is shown in Figure 9. Values ranging from 2321–2587 mg GA/100 g d.m. were obtained, which resulted in TPC retention of 84.2–93.9%. In a previous study using the same maltodextrin carrier, TPC retention was obtained at a similar level (88.1–95.6%) [9]. In a comparative study using the carriers tapioca dextrin and potato maltodextrin [8], TPC retention ranged from 77.5–99.3% and 71.3–83.9%, respectively. For similar feed solution formulations (with the same carrier and in the same amount), Bednarska and Janiszewska-Turak [12] achieved lower TPC retention values (65.6–69.6%). Using gum arabic as a carrier, the same authors obtained much higher TPC retention values (87.4–97.5%) [12]. In the case of spray drying phenolic chokeberry extract, different carriers were also used, and the obtained TPC retention values were 85.29% for gum arabic [14], 44.25–99.57% for maltodextrin: skim milk powder [16], 43.77–96.77% for maltodextrin: whey protein concentrate [16], and 73–97% for maltodextrin and skim milk [15]. The presented studies indicate that carriers other than maltodextrin, alone or in combination with maltodextrin, are able to achieve higher levels of TPC retention. However, also maltodextrin itself, with other appropriately optimized process parameters, allowed the achievement of high TPC retention values. In this study, maltodextrin was selected due to its lower price compared to other carriers and the greatest popularity in industrial applications.

The increase in drying temperature caused a decrease in TPC value due to thermal degradation of polyphenols during spray drying. Similar relationships were observed in other reported studies of spray drying chokeberry [8,9], as well as other berries [34,35]. Vidović et al. [17] described the same decreasing trend at drying temperatures above 140 °C, whereas below 140 °C, an increasing trend was observed. This could be due to very low inlet air temperatures, at which a greater amount of product was retained on the walls of the dryer, leading to an extended exposure time of the powder to hot air. This in turn promoted higher TPC losses despite the lower temperature [8]. However, there are also studies that have shown no statistically significant effect for chokeberry juice [12] and calafate berry juice [36], as well as an opposite (increasing) trend, such as for maqui berry extract [27].

My study also analyzed the relationship between TPC and SEC. The results showed that the most favorable inlet air temperature to achieve the highest TPC content (2587 mg GA/100 g) was at 150 °C. However, the most favorable temperature to minimize energy consumption was at 170 °C (SEC_min_ = 8364 kJ/kg H_2_O). Increasing the drying temperature from 150 to 170 °C lowered TPC by 2.9%, but caused a significant reduction in SEC (by 20.5%). Higher drying temperatures already caused dramatic decreases in TPC content and, at the same time, higher SEC values. Hence, in order to optimize both parameters, the most advantageous inlet air temperature was determined as 170 °C, which required minimum energy consumption without significantly increasing loss of bioactive properties.

**Figure 10:**
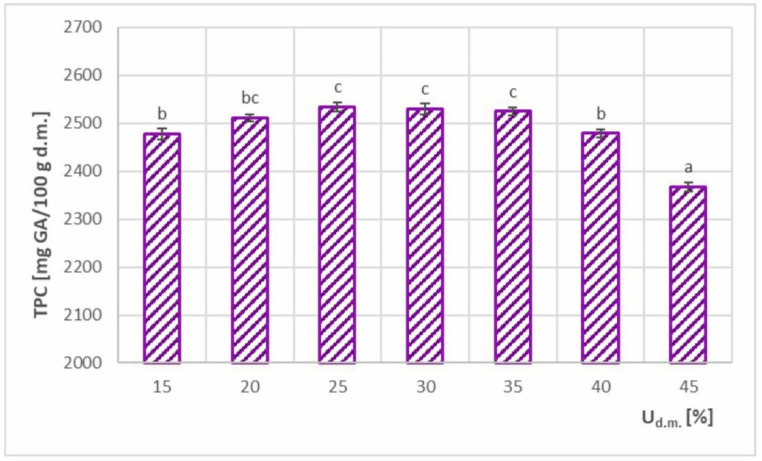
Total polyphenol content (TPC) in spray-dried chokeberry juice with different dry matter content in the feed solution (U_d.m._); (a, b, c—different letters show significant differences between mean values (*p* ≤ 0.05)).

Figure 10 shows TPC with different dry matter contents in the feed solution. Values ranging from 2367–2534 mg GA/100 g d.m. were obtained, resulting in TPC retention of 85.9–91.9%. The highest values were recorded in the range of 25–35% d.m., which were considered the optimal range. Taking into account that minimum SEC was achieved at a 35% d.m. concentration of the feed solution (Figure 8), it was advantageous for both SEC and TPC. Lower TPC values in U_d.m._ < 25% range were most likely related to the smaller particles formed in the drying solutions at lower concentration [28,37], which allowed more material–air contact surface and a higher ratio of drying air to dried material weight. Both factors may contribute to greater thermal degradation of polyphenols contained in chokeberry juice. For U_d.m._ ≥ 35% d.m., a statistically significant decreasing relationship was observed owing to the tendency to stick to the dryer walls at higher feed solution concentrations [38]. The powder deposited on and falling from the wall of the dryer over time was exposed to a longer residence time at higher temperature, which resulted in enhanced polyphenol degradation.

### 3.6. Anthocyanin Content (AC) and AC–SEC Relation

**Figure 11:**
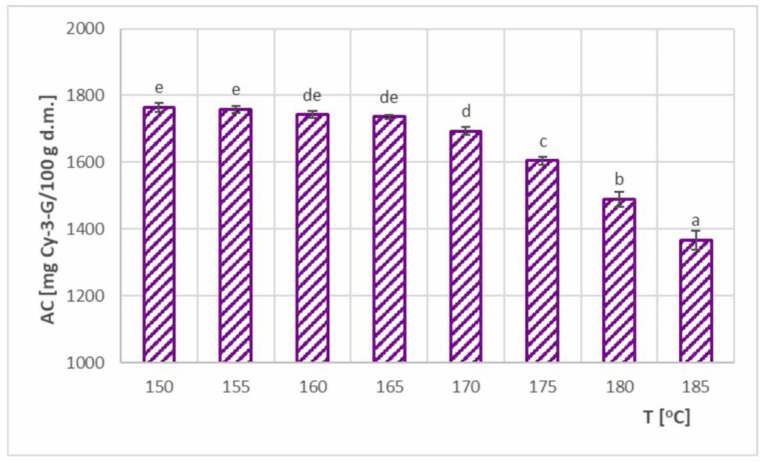
Anthocyanin content (AC) in spray-dried chokeberry juice at different inlet air temperatures (T); (a, b, c, d, e—different letters show significant differences between mean values (*p* ≤ 0.05)).

Anthocyanin content (AC) at various inlet air temperatures (T) is shown in Figure 11. Values ranging from 1367–1764 mg Cy-3-G/100 g d.m. were obtained, which promoted AC retention at 64.1–82.7%.

As in the case of TPC, the observed dependence of AC on the inlet air temperature generally decreased, where two ranges with different variability were clearly visible. In the temperature range of 150–170 °C, a slight decrease was observed, with a statistically significant decrease occurring in the range of 165–170 °C. Drying temperatures above 170 °C caused significant AC losses, which were much higher than for TPC. A similar observation was noted by Bednarska and Janiszewska-Turak [12], who examined drying chokeberry juice using maltodextrin as a carrier. In their study, the inlet air temperatures were tested in the range of 160–200 °C, and lower AC retention rates of 47.5–53.6% were obtained. In the case of spray drying of calafate berry [36], no statistically significant influence of temperature was obtained, while Bastías-Montes et al. [27] observed a growing trend for maqui berry extract. As with TPC retention, carriers other than maltodextrin can achieve higher AC retention values. Ćujić-Nikolić et al. [14] achieved 83.78% AC retention by using gum arabic as a carrier in the spray drying of phenolic chokeberry extract. Pieczykolan and Kurek [18] also investigated the spray drying of phenolic chokeberry extract by testing various additives to maltodextrin carrier (guar gum, gum arabic, pectin, beta-glucan and inulin). AC retention values between 78.61 and 92.98% were obtained. On the other hand, 82.7% AC retention was achieved using a maltodextrin carrier in the present study, and as Ćujić-Nikolić et al. [15] achieved an even higher value (96%) for this carrier, it can be concluded that maltodextrin can be a very effective carrier in the spray drying of chokeberry juices and extracts.

A comparison of AC retention results and SEC results showed a relationship similar to that with TPC. In the temperature range of 150–170 °C, the maximum differences in AC retention were 3.3% (at 150 °C), where the SEC differences were much greater, reaching up to 20.5%. Therefore, increasing the drying temperature from 150 °C to 170 °C was more favorable, as it significantly reduced energy consumption (by 20.5%) and slightly increased AC loss (by 3.3%). In contrast, at 170–185 °C, SEC only increased by 4.5%, while AC decreased by 19.3%. Therefore, increasing the inlet air temperature above 170 °C during spray drying of chokeberry juice was ineffective. The conducted analysis of the TPC–SEC relationship showed that the most favorable drying temperature in terms of AC–SEC was at 170 °C.

**Figure 12:**
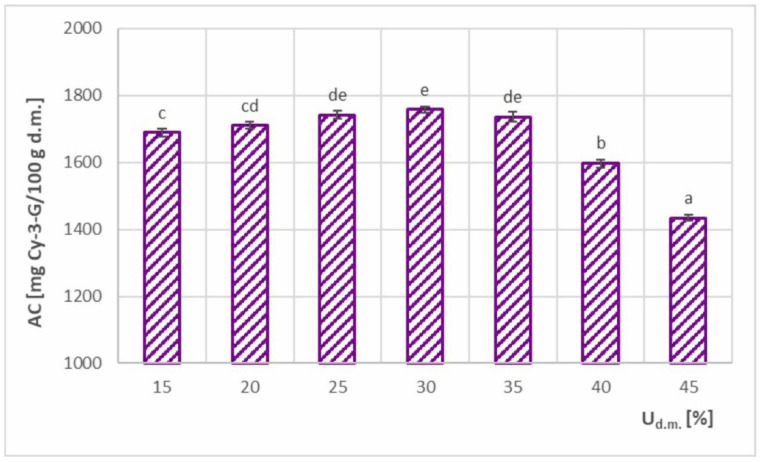
Anthocyanin content (AC) in spray-dried chokeberry juice with different dry matter content in the feed solution (U_d.m._); (a, b, c, d, e—different letters show significant differences between mean values (*p* ≤ 0.05)).

Figure 12 shows the AC of feed solutions with different dry matter contents. The obtained values ranged from 1245–1758 mg Cy-3-G/100 g d.m., which resulted in AC retention of 58.3–82.4%. As in the case of the relationship AC = f (T), the relationship AC = f (U_d.m._) also had two intervals with significantly different courses. In the range of 15–35% d.m., moderate variability was observed with fluctuations in AC value up to 4.1%, whereas in the range of 35–45% d.m., a decisive decrease occurred, with the maximum difference in AC values detected at 28.1%. The comparisons of the aforementioned ranges in relation to TPC = f (U_d.m._) and analysis of the efficiency of the process in terms of maximizing AC retention and minimizing SEC energy consumption revealed trends that were similar to those of the TPC–SEC relationship. In the range of 25–35% d.m. in the feed solution, the determined AC values were similar and were considered the highest. According to the SEC results presented in Figure 8, the range of maximum AC values coincided with the range of minimum SEC values (at U_d.m._= 35% d.m.). Hence, these value ranges were the most advantageous in terms of both energy consumption and AC.

Spray-dried chokeberry powders for U_d.m._ < 25% gave consequently lower AC values, owing to the similar mechanism described for TPC. A larger material–air contact area and higher ratio of drying air to the amount of dried material may also result in greater thermal degradation of AC in the chokeberry juice.

When spray drying chokeberry juices with higher feed solution concentrations (U_d.m._ ≥ 35% d.m.), a statistically significant decrease was observed. The observed trend was comparable to that of TPC, but it was clearly visible that AC was more sensitive to temperature due to much higher reduction in the AC value. In the case of TPC, a maximum decrease of 9.1% was observed, whereas that of AC was 28.3%. The mechanism of bioactive compound loss in both cases was analogous and associated with a greater tendency of the powder to stick to the dryer walls at higher feed solution concentrations (Section 3.5).

## 4. Conclusions

The production of chokeberry powder, which is an important functional food additive, should focus both on maximization of bioactive compound content and minimization of energy consumption. In the presented research range of inlet air temperatures (T = 150–185 °C) and feed solution concentrations (U_d.m._ = 15–45% d.m.), powders with a satisfactory moisture content below 3.22% were obtained. The most advantageous ranges of inlet air temperature and feed solution concentration in terms of PY were as follows: T = 160–170 °C and U_d.m._ = 25–35% d.m., where a powder yield of 91.6–94.4% was achieved. SEC in the spray drying process also showed the same optimum range as the drying temperature, i.e., T = 160–170 °C, whereas the feed solution concentration range shifted towards higher values, reaching the minimum at U_d.m._ = 35% d.m. Bioactive properties expressed in TPC and AC showed similarly effective ranges of drying parameters, which shifted towards lower values of T and U_d.m._ than those for SEC. The highest levels of TPC and TA were obtained at T = 150 °C and feed solution concentration in the range U_d.m._ = 25–30% d.m. The analysis of the SEC versus TPC and SEC versus AC relationships showed that the most favorable drying parameters for chokeberry juice were as follows: inlet air temperature T = 170 °C and feed solution concentration U_d.m._ = 35%. Chokeberry powders were achieved with approx. 3% lower retention of bioactive properties in relation to the maximum values, but with 20.5% lower SEC. The results of this research may be helpful in attempts to reduce the production costs for chokeberry powder as a valuable ingredient of instant functional food (while maintaining a high antioxidant potential).

## Data Availability

All data are contained within the article.
